# Genome-wide identification and expression analysis of the bHLH transcription factor family and its response to abiotic stress in sorghum [*Sorghum bicolor* (L.) Moench]

**DOI:** 10.1186/s12864-021-07652-9

**Published:** 2021-06-05

**Authors:** Yu Fan, Hao Yang, Dili Lai, Ailing He, Guoxing Xue, Liang Feng, Long Chen, Xiao-bin Cheng, Jingjun Ruan, Jun Yan, Jianping Cheng

**Affiliations:** 1 College of Agriculture, Guizhou University, Huaxi District, Guiyang City, 550025 Guizhou Province P.R. China; 2 Chengdu Food and Drug Inspection Institute, Chengdu, 610000 P.R. China; 3 Department of Nursing, Sichuan Tianyi College, Mianzhu, 618200 P.R. China; 4 Department of Environmental and Life Sciences, Sichuan MinZu College, Kangding, 626001 P.R. China; 5 School of Pharmacy and Bioengineering, Chengdu University, Chengdu, 610106 P.R. China

**Keywords:** *Sorghum bicolor*, bHLH gene family, Genome-wide analysis, Abiotic stress

## Abstract

**Background:**

Basic helix-loop-helix (bHLH) is a superfamily of transcription factors that is widely found in plants and animals, and is the second largest transcription factor family in eukaryotes after MYB. They have been shown to be important regulatory components in tissue development and many different biological processes. However, no systemic analysis of the bHLH transcription factor family has yet been reported in *Sorghum bicolor*.

**Results:**

We conducted the first genome-wide analysis of the bHLH transcription factor family of *Sorghum bicolor* and identified 174 *SbbHLH* genes. Phylogenetic analysis of SbbHLH proteins and 158 *Arabidopsis thaliana* bHLH proteins was performed to determine their homology. In addition, conserved motifs, gene structure, chromosomal spread, and gene duplication of *SbbHLH* genes were studied in depth. To further infer the phylogenetic mechanisms in the *SbbHLH* family, we constructed six comparative syntenic maps of *S. bicolor* associated with six representative species. Finally, we analyzed the gene-expression response and tissue-development characteristics of 12 typical *SbbHLH* genes in plants subjected to six different abiotic stresses. Gene expression during flower and fruit development was also examined.

**Conclusions:**

This study is of great significance for functional identification and confirmation of the *S. bicolor bHLH* superfamily and for our understanding of the *bHLH* superfamily in higher plants.

**Supplementary Information:**

The online version contains supplementary material available at 10.1186/s12864-021-07652-9.

## Background

Transcription factors (TFs) play an important role in controlling plant growth and environmental adaptation [1, 2]. They regulate gene expression by combining with specific cis-promoter elements that specifically regulate certain genes or transcription rates, thereby playing a unique regulatory role in plant morphogenesis, cell-cycle processes, and the like [3, 4]. Structurally, the typical TF includes a DNA-binding site, a transcription-activation or repression domain, an oligomerization site, and a nuclear-localization site. TF genes, such as members of the *bHLH*, *WRKY*, *MYB*, *bZIP* and other TF families, constitute a high proportion of all plant genomes, and their target genes are widely involved in physiological processes, such as plant development and stress responses [5, 6].

Basic helix-loop-helix (bHLH) is a superfamily of TFs that is widely found in plants and animals; it is the second largest TF family among eukaryotic proteins after MYB [7, 8]. The first discovered *bHLH* family member was the c-myc proto-oncogene of avian myeloid cell carcinoma virus [9]. The bHLH TFs are so named because of their structural feature of a bHLH domain in all family members. The amino acid sequence of this domain is highly conserved. There are about 50 to 60 amino acid residues that can be divided into two regions based on their functions: a basic region and the HLH [9, 10]. The basic domain is located at the N terminus of the conserved domain of bHLH and contains about 15 amino acids. It can bind to the cis-acting element E-box (5′-canntg-3′). Therefore, the number of basic and key amino acid residues in the basic region determine whether the bHLH TF has DNA-binding activity. The HLH domain is distributed at the C terminus of the gene sequence, where two α-helices are connected by a low-conserved loop, which is essential for the formation of homodimers or heterodimers of bHLH TFs [11, 12, 13]. Based on their ability to bind DNA, bHLH TFs can be divided into two categories: DNA binding and non-DNA binding. These can be further divided into E-box binding and non-E-box binding. The most common method of E-box binding is G-box binding (5′-cacgtg-3′) [10, 14, 15]. According to Atchley et al. [10, 16], Glu and Arg at positions 9 and 13 of the basic region, namely E9 and R13, are essential amino acid residues that bind to E-box and H/K5-E9-R13 patterns, and bind to G-box. The study of *bHLH* gene family in different species will help to understand the evolutionary process and biological function. Previous phylogenetic results showed that *bHLH* proteins in plants were divided into 26 subfamilies, 20 of which were found in the common ancestor of vascular and bryophytes plants [17]. Toledo Ortiz et al. [15] divided 147 *AtbHLH* proteins into 21 subfamilies; and Li et al. [18] divided 167 *OsbHLH* proteins into 22 subfamilies.

The bHLH TF family is involved in plants’ perception of the external environment, cell-cycle regulation, and tissue differentiation [18, 19]. Different subfamilies regulate different biological processes, such as transduction of light signals [20, 21] and hormone signals [22, 23], and organ development [24–26, 27]. Under stress conditions, certain bHLH TFs are activated; they combine with the promoters of key genes involved in various signaling pathways, and regulate the transcription level of these target genes, thereby regulating the plants’ stress tolerance. For example, some researchers have found that the homologous *bHLH* genes *bhlh068* of *Oryza sativa* and *bHLH112* of *Arabidopsis thaliana* play an active role in the response to salt stress, but have opposite effects on regulation of plant flowering [28]. Appropriate TFs, together with *AtbHLH38* and *AtbHLH39*, can regulate iron metabolism in *Arabidopsis* [29]. *Atbhlh112* is a transcriptional activator of drought and other stress signal-transduction pathways, but it has an inhibitory effect on root development [30]. In *Nicotiana tabacum*, plants overexpressing *Ntbhlh123* have enhanced resistance under low-temperature stress [31]. bHLH TFs are involved in regulating the accumulation of secondary metabolites in plants [32]. These examples all show the roles of bHLH TFs in the plant response to stress.

The expansion of this family is closely related to plant evolution and diversity [33, 34], not only in higher plants, but also in lower plants or non-plants, such as algae, mycobacteria, lichens and mosses [34]. With regards to abiotic stresses, *bHLH* is mainly involved in the defense responses to drought, high temperature, low temperature, and high salinity, which are unique to the terrestrial environment. Therefore, the evolution of the *bHLH* gene family provides clues to understanding the evolution of green algae to flowering plants through their adaptation to environmental changes. In particular, genome-wide analysis of *bHLH* gene families of different species will help understand the biological function and evolutionary origin of the *bHLH* genes.

*Sorghum bicolor* (L.) Moench is an annual row crop in the family Gramineae [35]. It is a common grain crop, which is used to produce food and beverage, widely distributed in the tropical, subtropical and temperate regions of the world and cultivated in the northern and southern provinces of China. *S. bicolor* seeds serve as a food source in China, North Korea, the former Soviet Union, India and Africa [36]. *S. bicolor* has rich genetic and phenotypic diversity, especially in plant height, seed color, seed size and branch number. Moreover, *S. bicolor* is a particularly nutritious crop, high in resistant starch, proteins, vitamins and polyphenols [37, 38], and it is widely used in the brewing industry [39]. In the long-term environmental adaptation, different varieties have been formed on sorghum, and some extreme abiotic stresses still have significant effects on its growth and development. For example, *S. bicolor* plants show reduced floret fertility and single-grain weight under high temperature, thereby reducing yield [40, 41]; low temperature leads to weakening of this crop’s growth potential, and plants are generally seriously damaged by frost [42]. *S. bicolor* has a well-developed root system that enables it to survive drought to some extent [43, 44]; nevertheless, long-term extreme drought has a huge impact on growth and yield [43]. In the process of *S. bicolor* production, pests, diseases, weeds and other biotic stresses will also cause serious yield losses [44]. Because *S. bicolor* is cultivated throughout the world, it has great economic and research value, and the identification of its functional genes is important.

In 2009, the completion and publication of the whole *S. bicolor* genome sequence enabled us to further explore, clone and verify the *bHLH* genes related to its stress resistance [45]. The *S. bicolor* genome is 750 Mb in length, with about 30,000 genes, ca. 75% more than in rice [46]. The *bHLH* gene family has been widely studied in many plant species, such as *Arabidopsis* [15], rice [18], Chinese cabbage [26], tomato [47], common bean [48], apple [49], peanut [50], *Brachypodium distachyon* [51], potato [52], maize [53], wheat [54], MOSO bamboo [55], *Carthamus tinctorius* [56], Chinese jujube [57], pepper [58], Jilin ginseng [59], pineapple [60], and tartary buckwheat [61], among others. However, at present, our understanding of gene families in *S. bicolor* is very limited. The main gene families identified in this plant are MADS-box [62], Dof [63], CBL [64], ERF [65], SBP-box [66], HSP [67], LEA [68], and NAC [69], among others. Because *bHLH* genes play an important role in various physiological processes, it is of great significance to systematically study the bHLH family in *S. bicolor*. Here, we identified 174 *bHLH* genes in *S. bicolor* and classified them into 24 major groups. Exon–intron structure, motif composition, gene duplication, chromosome distribution, and phylogeny were analyzed. The expression of *bHLH* family members in *S. bicolor* under different biological processes and abiotic stresses was also analyzed. This study provides valuable clues to the functional identification and evolutionary relationships of *S. bicolor.*

## Results

### Identification of *bHLH* genes in *S. bicolor*

To identify all possible *bHLH* members in the *S. bicolor* genome, we used two BLAST methods (Additional file 1: Table S1). To better distinguish these genes, we named them *SbbHLH001* to *SbbHLH174* according to their location on the *S. bicolor* chromosomes (Additional file 1: Table S1) and provide the genes’ characteristics, including molecular weight, isoelectric point (pI), protein length, domain information, and subcellular localization (http://cello.life. nctu.edu.tw/) (Additional file 1: Table S1).

Of the 174 SbbHLH proteins, SbbHLH031 and SbbHLH168 were the smallest with 87 amino acids, and the largest protein was SbbHLH040 with 1105 amino acids. The molecular mass of the proteins ranged from 9.67 kDa (SbbHLH168) to124.74 kDa (SbbHLH040), and the pI ranged from 4.53 (SbbHLH081) to 12.05 (SbbHLH004), with a mean of 6.70. Of all of the *SbbHLH* genes, 14 contained the bHLH-MYC-N domain and 172 contained the HLH domain (the exceptions being *SbbHLH097* and *SbbHLH116*). The predicted subcellular localization results showed that 141 SbbHLHs are located in the nucleus, 26 in the cytoplasm, 4 in the mitochondria, 2 (SbbHLH103 and SbbHLH090) in the endoplasmic reticulum, and 1 (SbbHLH095) in the cytoskeleton (Additional file 1: Table S1). The ratio of *SbbHLH* genes to total genes in the *S. bicolor* genome was about 0.58%, which is similar to *Arabidopsis* (0.59%), but more than in rice (0.44%) [18], poplar (0.40%) [27], and tomato (0.46%) [48].

### Multiple sequence alignment, phylogenetic analysis, and classification of *SbbHLH* genes

**Fig. 1:**
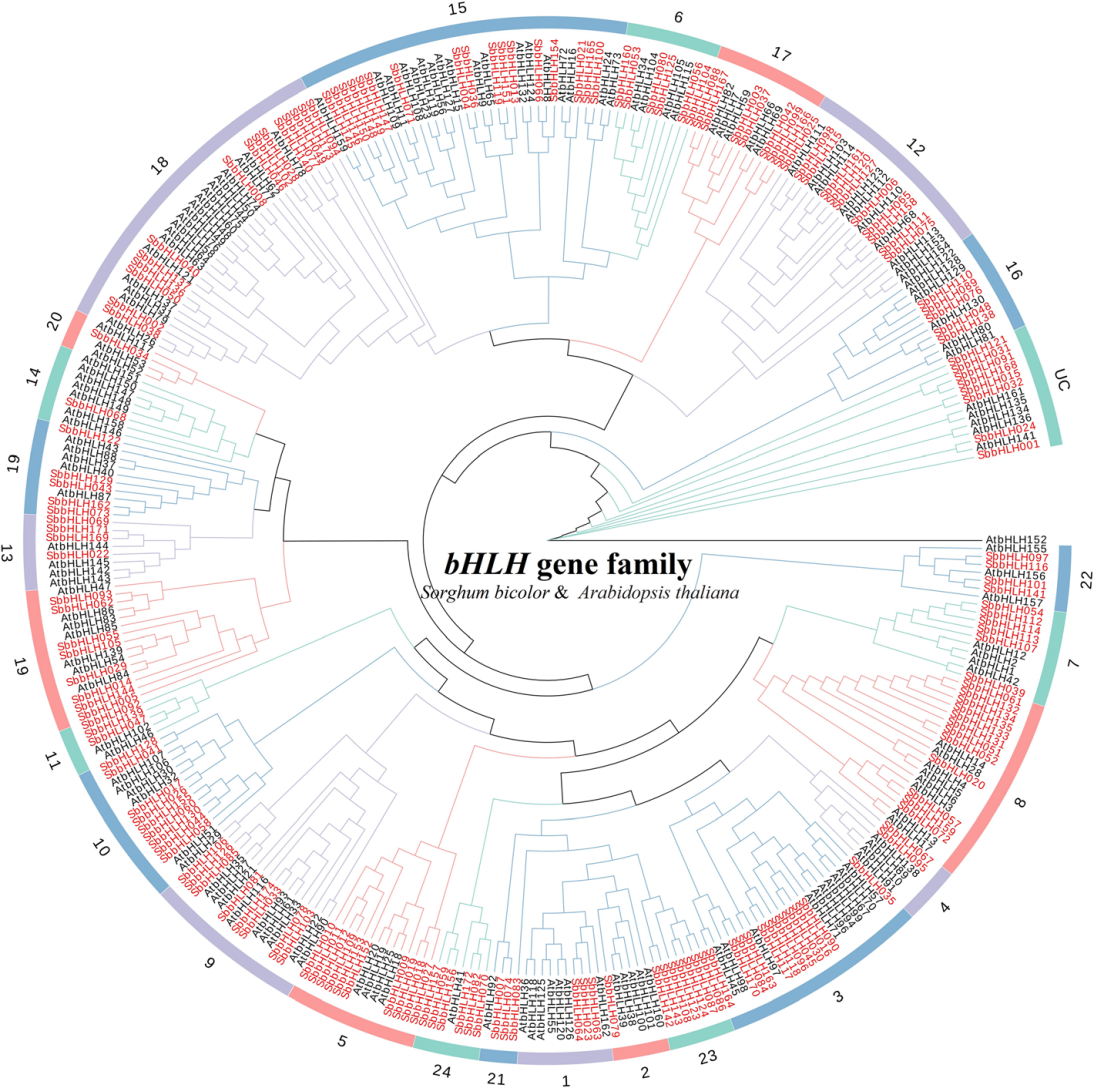
Unrooted phylogenetic tree showing relationships among bHLH domains of *S. bicolor* and *Arabidopsis*. The phylogenetic tree was derived using the NJ method in MEGA7.0. The tree shows the 24 phylogenetic subfamilies and 1 unclassified group (UC) marked with red font on a white background. bHLH proteins from *Arabidopsis* are marked with the prefix ‘At’

We constructed a phylogenetic tree using the neighbor-joining (NJ) method with a bootstrap value of 1000 based on the amino acid sequences of 174 SbbHLH and 158 AtbHLH proteins (Fig. 1; Additional file 1: Table S1). According to the topological structure of the tree and classification method proposed by Pires and Gabriela [15, 17], 332 *bHLH* genes in the phylogenetic tree were divided into 24 clades (groups 1–24) and 1 orphan [1, 6, 7]. The unclassified group (UC) contained 8 *SbbHLH* and 6 *AtbHLH* genes, and 149 SbbHLH proteins clustered into 21 subfamilies. This is consistent with the taxonomic group of *bHLH* proteins in *Arabidopsis* [18], indicating no loss of those proteins during the long-term evolution in *S. bicolor* evolution. Seventeen *S. bicolor* proteins constituted three typical topological structures (groups 22–24), suggesting that these are new characteristics in the evolution of *S. bicolor* diversity. None of AtbHLHs was assigned into subfamily 23,which contained 7 SbbHLHs (SbbHLH86, SbbHLH87, SbbHLH108, SbbHLH123, SbbHLH124, SbbHLH142, SbbHLH143); this group might indicate a new evolutionary direction for *S. bicolor*. Among the 24 subfamilies, the subfamily 15 had the largest number of members (17 SbbHLHs), and subfamilies 2 (SbbHLH79), 14 (SbbHLH68), and 20 (SbbHLH34) had the fewest (1 SbbHLH). Eight *SbbHLH* genes, which are not clearly classified into any subfamily, were classified as “orphans” [15, 16] (Fig. 1, Additional file 1: Table S1). A phylogenetic tree for *Arabidopsis* showed that some *SbbHLH*s are tightly grouped with the *AtbHLH*s (bootstrap support ≥70). These may be orthologous to the *AtbHLH*s and have similar functions.

**Fig. 2:**
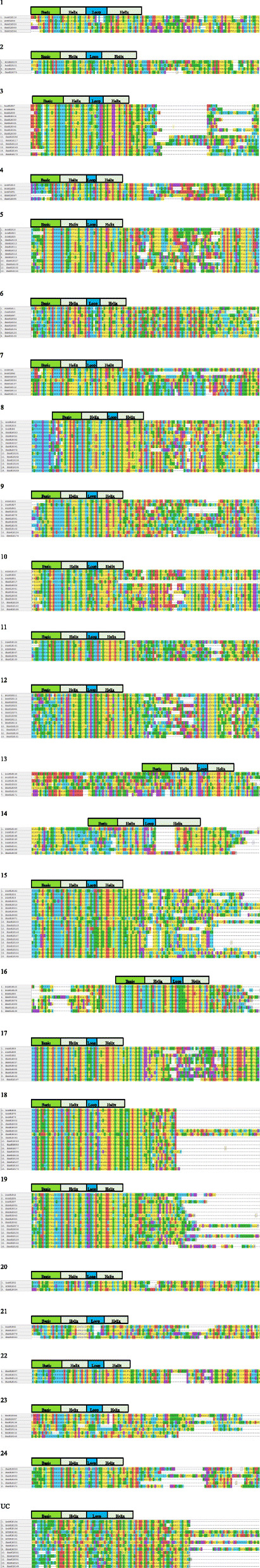
Multiple sequence alignment of the bHLH domains of the members of 24 phylogenetic subfamilies and 1 unclassified group (UC) of the SbbHLH protein family. The scheme at the top depicts the locations and boundaries of the basic, helix, and loop regions in the bHLH domain

The bHLH domain of *Arabidopsis* bHLH proteins and those from subgroups 1–21 were randomly selected as representatives of groups and subgroups for further multiple-sequence comparison (Fig. 2, Additional file 1: Table S1). The *SbbHLH* members from groups 22–24 were selected for the comparison. The bHLH domains of *S. bicolor* span approximately 50 amino acids. As shown in Fig. 2, although the characteristic bHLH domain is well conserved in *Arabidopsis* and *S. bicolor*, the regions outside of this domain in the rest of the protein are usually differentiate and diversify [13, 14, 18]. We considered the basic region to be 17 amino acids long based on Gabriela’s view [15]. In terms of amino acid structure, the loop was the most divergent region of this domain, especially in subfamily 6, 10 and 23, as has been observed for bHLH proteins from other plants, including *Arabidopsis* [18], potato [26], tomato [48] and buckwheat [61].

### Conserved motifs and gene structure analysis of *SbbHLH* genes

**Fig. 3:**
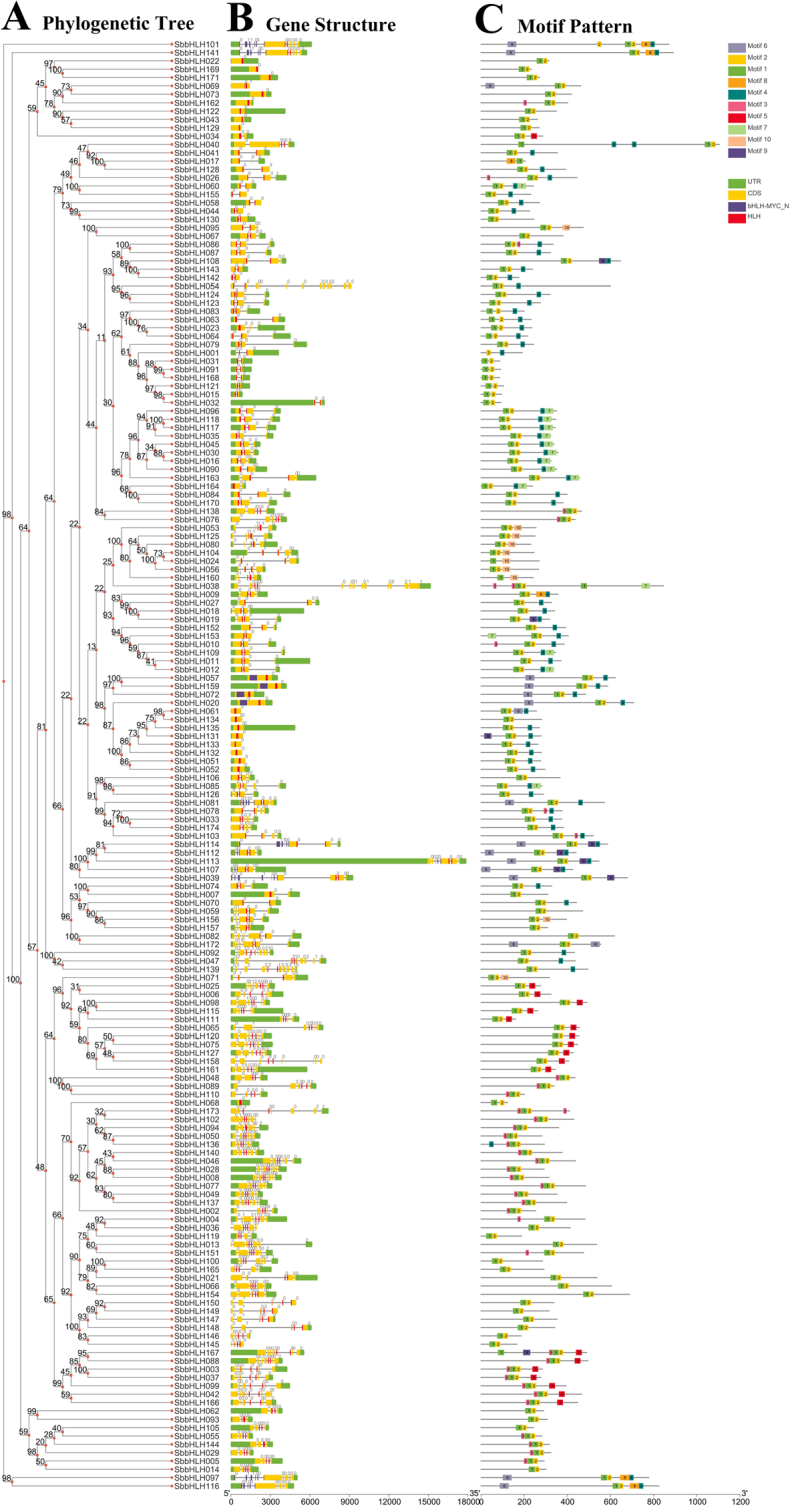
Phylogenetic relationships, gene-structure analysis, and motif distributions of *S. bicolor bHLH* genes. **a** Phylogenetic tree was constructed by the NJ method with 1000 replicates on each node. **b** Exons and introns are indicated by yellow rectangles and gray lines, respectively. **c** Amino acid motifs in the SbbHLH proteins (1–10) are represented by colored boxes. The black lines indicate relative protein lengths

To understand the structural components of the *SbbHLH* genes, their exon and intron structures were obtained by comparing the corresponding genomic DNA sequences (Fig. 3, Additional files 1and 2: Tables S1 and S2). A comparison of the number and position of the exons and introns revealed that the 174 *SbbHLH* genes had different numbers of exons, varying from 1 to 12 (Fig. 3a/b). In addition, 17 (9.77%) genes contained 1 exon, and the remaining genes had 2 or more exons. The 17 intronless genes belonged to four subfamilies (8, 13, 14, 19), but were mainly in subfamilies 8 and 19. The largest proportion of *SbbHLH* genes (*n* = 31) had 2 introns. *SbbHLH038* and *SbbHLH054* had the most introns, with 11. Group 1, 2, 4, 10, 20, 21 and 23 members contained 1 or 2 introns. Further analyses indicated that group 18 showed more diversity in the number of introns. In general, members of the same subfamily had similar gene structures.

To further study the characteristic region of the SbbHLH proteins, the motifs of 174 SbbHLH proteins were analyzed using the online tool MEME. A total of 10 distinct conserved motifs (motifs 1–10) were found (Fig. 3c, Additional file 2: Table S2). As exhibited in Fig. 3c, motifs 1 and 2 were widely distributed in the SbbHLHs, except for SbbHLH001 and SbbHLH017, and the two motifs were very close to each other in the bHLH proteins. SbbHLH members within the same groups were usually found to share a similar motif composition. For example, group 1, 2, 3, 5, 7, 9, 11 and 23 members contained motifs 1, 2, and 4; groups 12 and 17 contained motifs 1, 2, and 5; group 16 contained motifs 3, 1, and 2; and group 22 contained motifs 6, 1, 2, 8, and 4. At the same time, we found that some motifs were only present in specific subfamilies. In addition, motif 5 was specific to groups 12, 17 and 20, whereas motif 8 was specific to groups 5, 10 and 22. Further analysis showed that some of the motifs could only be distributed in specific locations of the pattern. For example, motif 1 was always distributed at the start of the pattern in groups 1, 2, 3, 4, 5, 6, 9, 10, 11, 12, 13, 14, 15, 20, 21, 23 and 24; motif 6 was almost always distributed at the start of groups 7 and 22; motif 3 was almost always distributed at the start of groups 16, 17 and 18. Motif 4 was almost always distributed at the end of the pattern in groups 1, 2, 7, 8, 9, 10, 11, 22 and 23; and motif 10 was distributed at the end of the pattern in the group 6. The functions of most of these conserved motifs remain to be elucidated. Overall, members that belonged to the same subfamily had similar gene structure and motif composition, in accordance with the results of the phylogenetic analysis, and supporting the reliability of the population classification.

### Chromosomal spread and gene duplication of *SbbHLH* genes

**Fig. 4:**
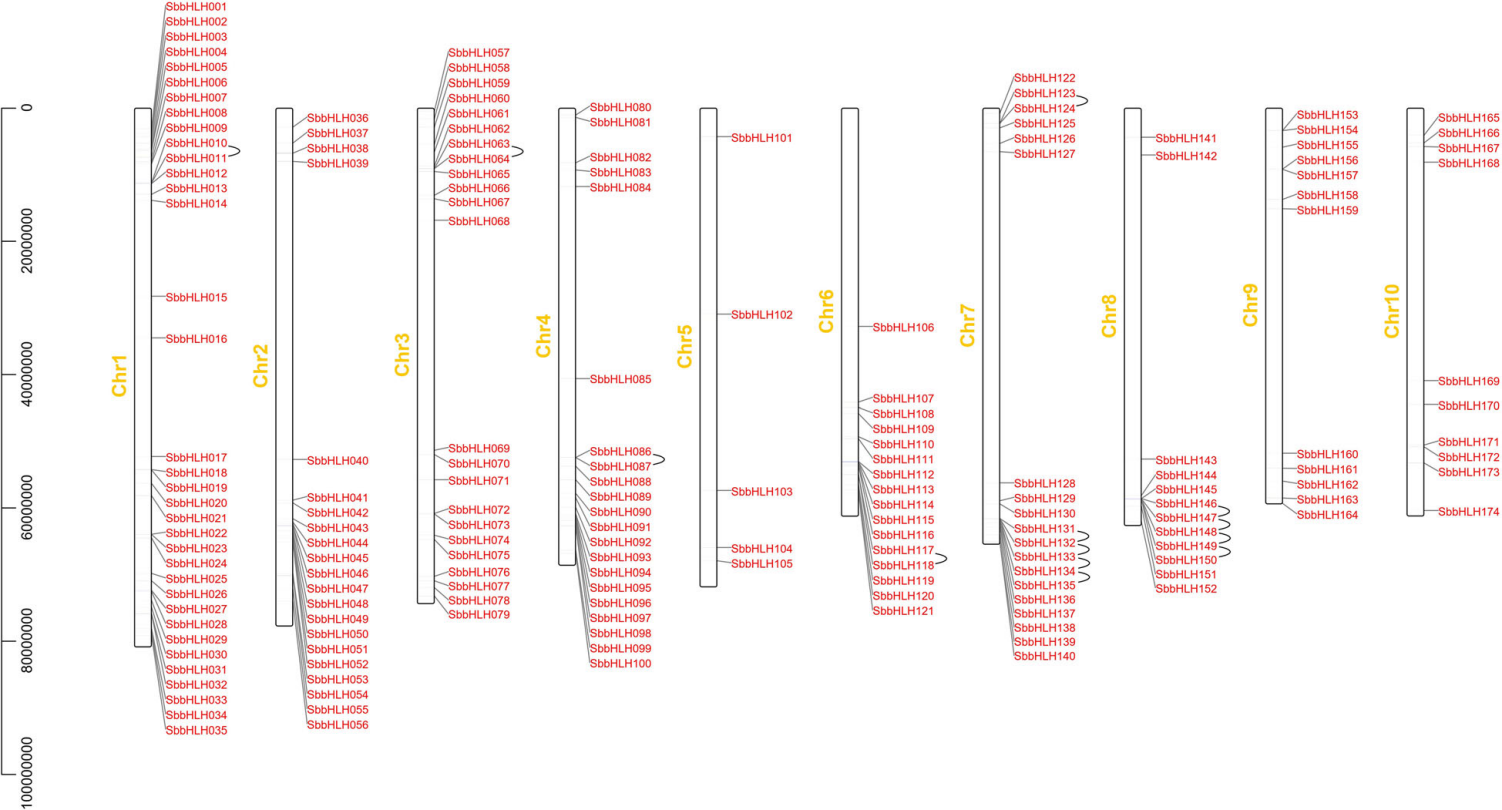
Schematic representation of the chromosomal distribution of the *S. bicolor bHLH* genes. Vertical bars represent the chromosomes of *S. bicolor*. The chromosome number is indicated to the left of each chromosome. The scale on the left represents chromosome length

A map of the physical position of the *SbbHLH* genes was created based on the latest *S. bicolor* genome database (Fig. 4, Additional file 3: Table S3). The distribution of the 174 *SbbHLH* genes on chromosomes (Chr) 1 to 10 was uneven (Fig. 4). Each of the *SbbHLH*s’ names was given according to its physical position from the top to the bottom on *S. bicolor* Chr1 to Chr10. Chr1 contained the largest number of *SbbHLH* genes (35 genes, ~ 20.11%), followed by Chr3 (23, ~ 13.22%), while Chr5 contained the least (5, ~ 2.87%). Chr2 and Chr4 each contained 21 (~ 12.07%) *SbbHLH* genes. Chr8 and Chr9 each contained 12 (~ 6.90%) *SbbHLH* genes. Chr6, Chr7, and Chr10 contained 16 (~ 9.20%), 19 (~ 10.92%), and 10 (~ 5.75%) *SbbHLH* genes, respectively. Interestingly, most *SbbHLH* genes were distributed at the ends of the 10 chromosomes. In addition, we observed a large number of *SbbHLH* gene-duplication events. A chromosomal region within 200 kb exhibiting two or more identical genomic regions is defined as a tandem duplication event [35]. On chromosomes 1, 3, 4, 6, 7 and 8, we discovered 13 tandem duplication events involving 20 *SbbHLH* genes (Fig. 4). *SbbHLH132*, *SbbHLH133*, *SbbHLH134*, *SbbHLH147*, *SbbHLH148* and *SbbHLH149* each had two tandem repeat events (*SbbHLH132* and *SbbHLH131* / *SbbHLH133*; *SbbHLH133* and *SbbHLH132* / *SbbHLH134*; *SbbHLH134* and *SbbHLH133* / *SbbHLH135*; *SbbHLH147* and *SbbHLH146* / *SbbHLH148*; *SbbHLH148* and *SbbHLH147* / *SbbHLH149*; *SbbHLH149* and *SbbHLH148* / *SbbHLH150*). All genes that formed tandem repeat events came from the same subfamily. For example, *SbbHLH117* and *SbbHLH118* were tandem repeat genes and they clustered together in subfamily 3 (Fig. 4, Additional file 3: Table S3).

**Fig. 5:**
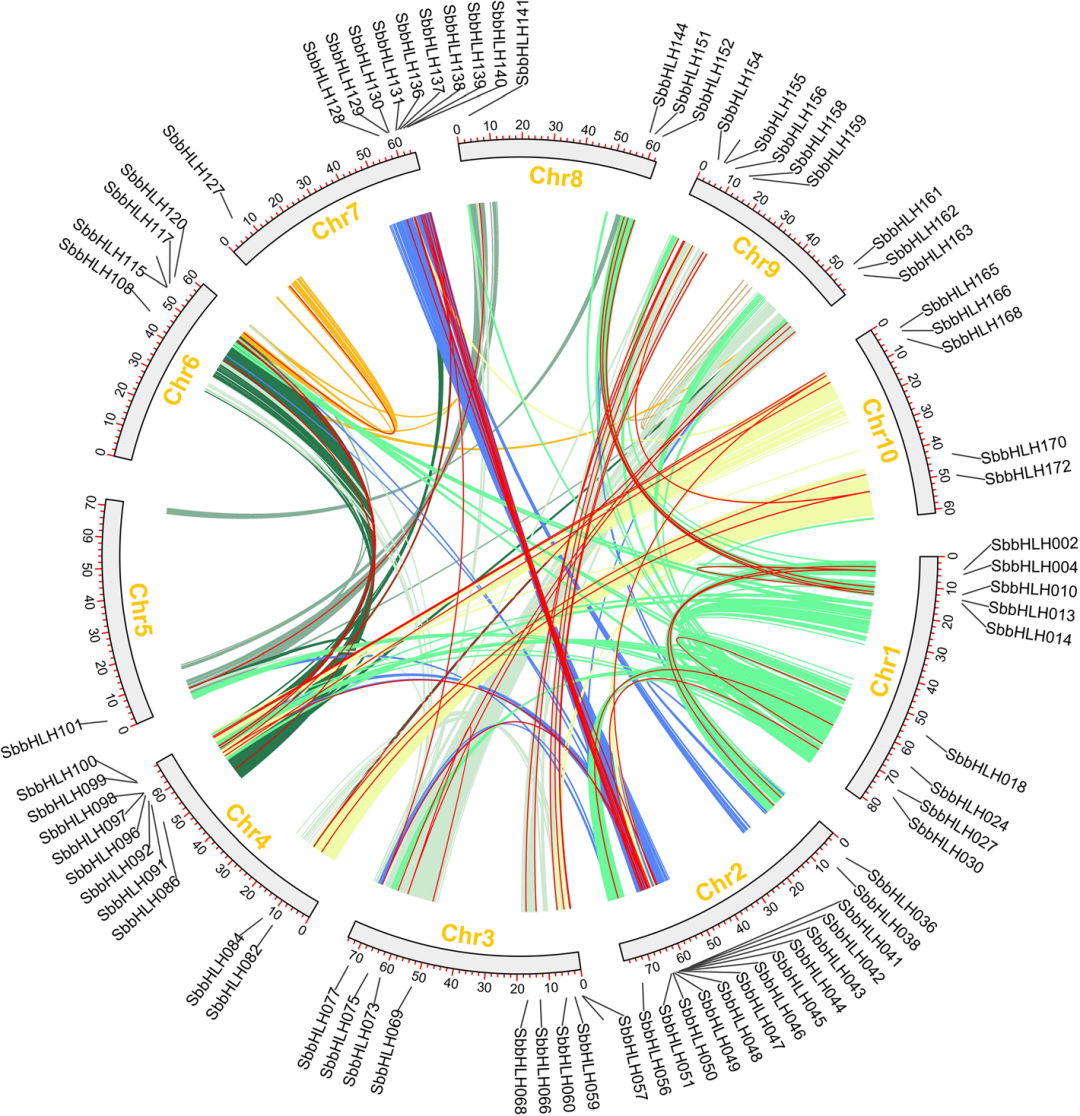
Schematic representation of the chromosomal distribution and interchromosomal relationships of *S. bicolor bHLH* genes. Colored lines indicate all synteny blocks in the *S. bicolor* genome and the red lines indicate duplicated *bHLH* gene pairs. Chromosome number is indicated at the bottom of each chromosome

In addition, there were 42 pairs of segmental duplications in the *SbbHLH* genes (Fig. 5, Additional file 4: Table S4). As shown in Figs. 5, 71 (40.8%) paralogs were identified in the *SbbHLH* gene family, indicating an evolutionary relationship among these *bHLH* members. The *SbbHLH* genes were unevenly distributed in 10 *S. bicolor* linkage groups (LGs) (Fig. 5). Some LGs had more *SbbHLH* genes than others (LG2, LG7). LG2 had the most *SbbHLH* genes (14), and LG5 had the least (1). Further analysis of the subfamilies of these genes showed that most of them are linked within their subfamily, except for *SbbHLH024* / UC and *SbbHLH056* / 6. For all identified *SbbHLH* genes, group 18 had the largest number of linked genes (9/71). In addition, the group 15 had 8 genes, while groups 13 and 6 had only 1 (Additional file 4: Table S4). These results suggest that some *SbbHLH* genes may have been produced by gene-replication events, and that these replication events played a major role in the occurrence of new functions in *S. bicolor* evolution and the amplification of the *SbbHLH* gene family.

### Synteny analysis of *SbbHLH* genes

**Fig. 6:**
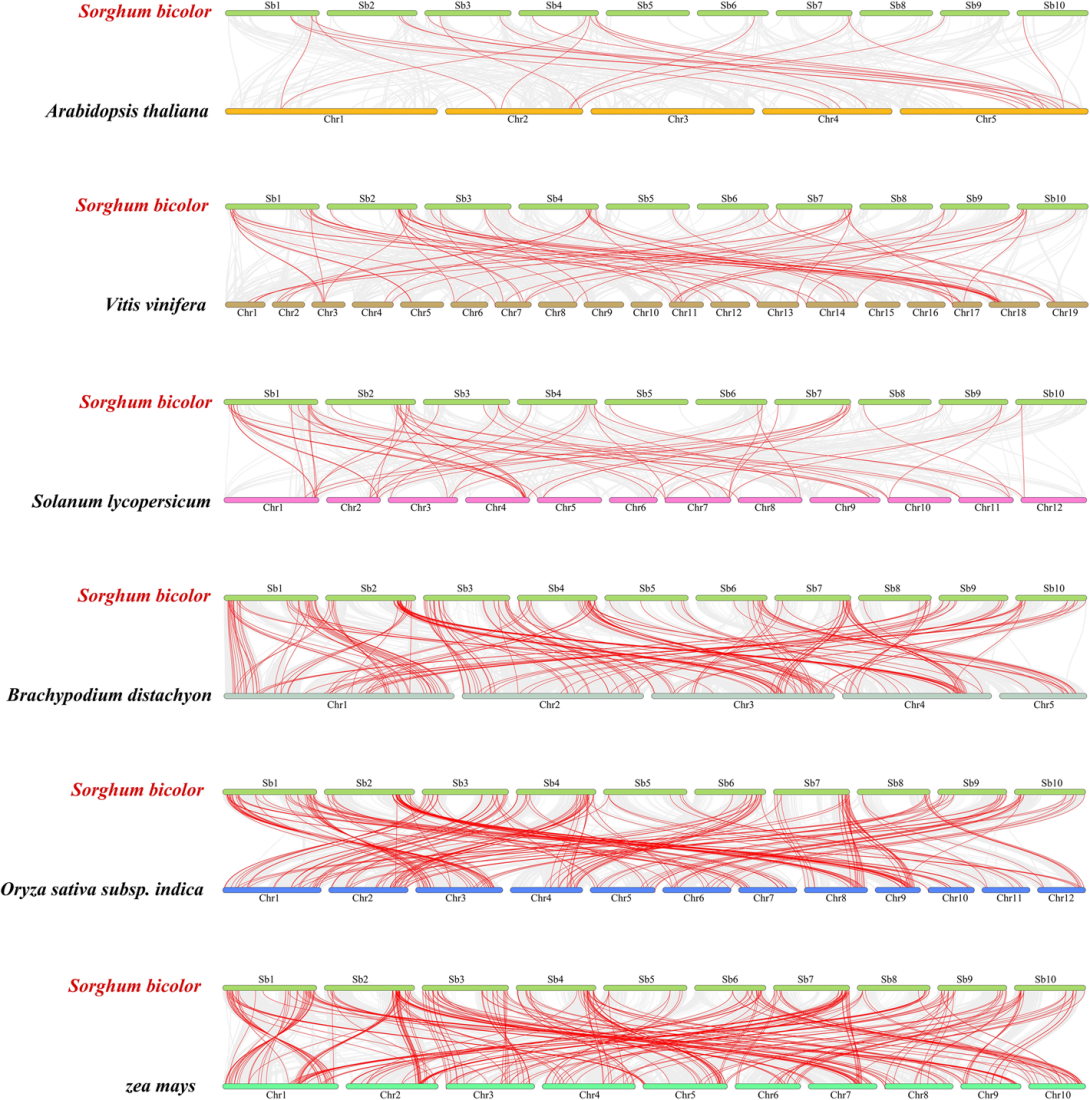
Synteny analyses of the *bHLH* genes between *S. bicolor* and six representative plant species (*Arabidopsis thaliana*, *Vitis vinifera*, *Solanum lycopersicum*, *Brachypodium distachyon*, *Oryza sativa subsp. indica*, *Zea mays*). Gray lines on the background indicate the collinear blocks in *S. bicolor* and other plant genomes; red lines highlight the syntenic *S. bicolor bHLH* gene pairs

To further infer the phylogenetic mechanisms of the *S. bicolor* bHLH family, we constructed six comparative synteny maps of *S. bicolor*’s association with six representative species, including three dicotyledons (*A. thaliana*, *Vitis vinifera* and *Solanum lycopersicum*) and three monocotyledons (*B. distachyon*, *O. sativa* and *Zea mays*) (Fig. 6, Additional file 5: Table S5). A total of 150 *SbbHLH* genes showed syntenic relationships with those in *A. thaliana* (16), *V. vinifera* (46), *S. lycopersicum* (37), *B. distachyon* (129), *O. sativa* (135) and *Z. mays* (195) (Additional file 5: Table S5). The numbers of orthologous pairs between the other six species (*A. thaliana*, *V. vinifera*, *S. lycopersicum*, *B. distachyon*, *O. sativa* and *Z. mays*) were 20, 66, 59, 194, 208 and 273, respectively. Some *SbbHLH* genes were associated with at least four syntenic gene pairs (particularly between *S. bicolor* and *Z. mays bHLH*), such as *SbbHLH043*, *SbbHLH049*, *SbbHLH050*, *SbbHLH101*, *SbbHLH137*, *SbbHLH138*, *SbbHLH141* and *SbbHLH166*, hinting at these genes’ important role during evolution.

As expected, some collinear gene pairs (with 57 *SbbHLH* genes) identified between *S. bicolor* and *B. distachyon*, *O. sativa* or *Z. mays* were not found between *S. bicolor* and *A. thaliana*, *V. vinifera*, or *S. lycopersicum*, such as *SbbHLH001* with KQK12528/BGIOSGA013800-TA/Zm00001d034596_T001, and *SbbHLH004* with KQK12892/BGIOSGA013672-TA/Zm00001d034298_T001. This suggests that these homologous genes may be gradually formed after the independent differentiation of monocotyledons (Additional file 5: Table S5). Similar patterns were also observed between *S. bicolor* and *O. sativa*/ *Z. mays*, which may be related to the phylogenetic relationships between *S. bicolor* and the other six plant species. In addition, some *SbbHLH* genes were found to be associated with at least one syntenic gene pair among the six plants (especially between *S. bicolor* and *Z. mays*), such as *SbbHLH030*, *SbbHLH045*, *SbbHLH050*, *SbbHLH066*, *SbbHLH099*, *SbbHLH136*, *SbbHLH138*, *SbbHLH154*, *SbbHLH166*, suggesting that these orthologous pairs already existed before the ancestral divergence, and thus indicating that these genes may have played an important role in the *bHLH* gene family during evolution. To better understand the evolutionary constraints of the *bHLH* gene family in *S. bicolor*, the *SbbHLH* genes were subjected to Tajima’s D Neutrality Test. Calculations gave D = 7.736378, the large deviation from 0, suggesting that the *SbbHLH* gene family might have experienced strong purifying selective pressure during evolution.

### Evolutionary analysis of the *SbbHLH* genes and *bHLH* genes of several different species

**Fig. 7:**
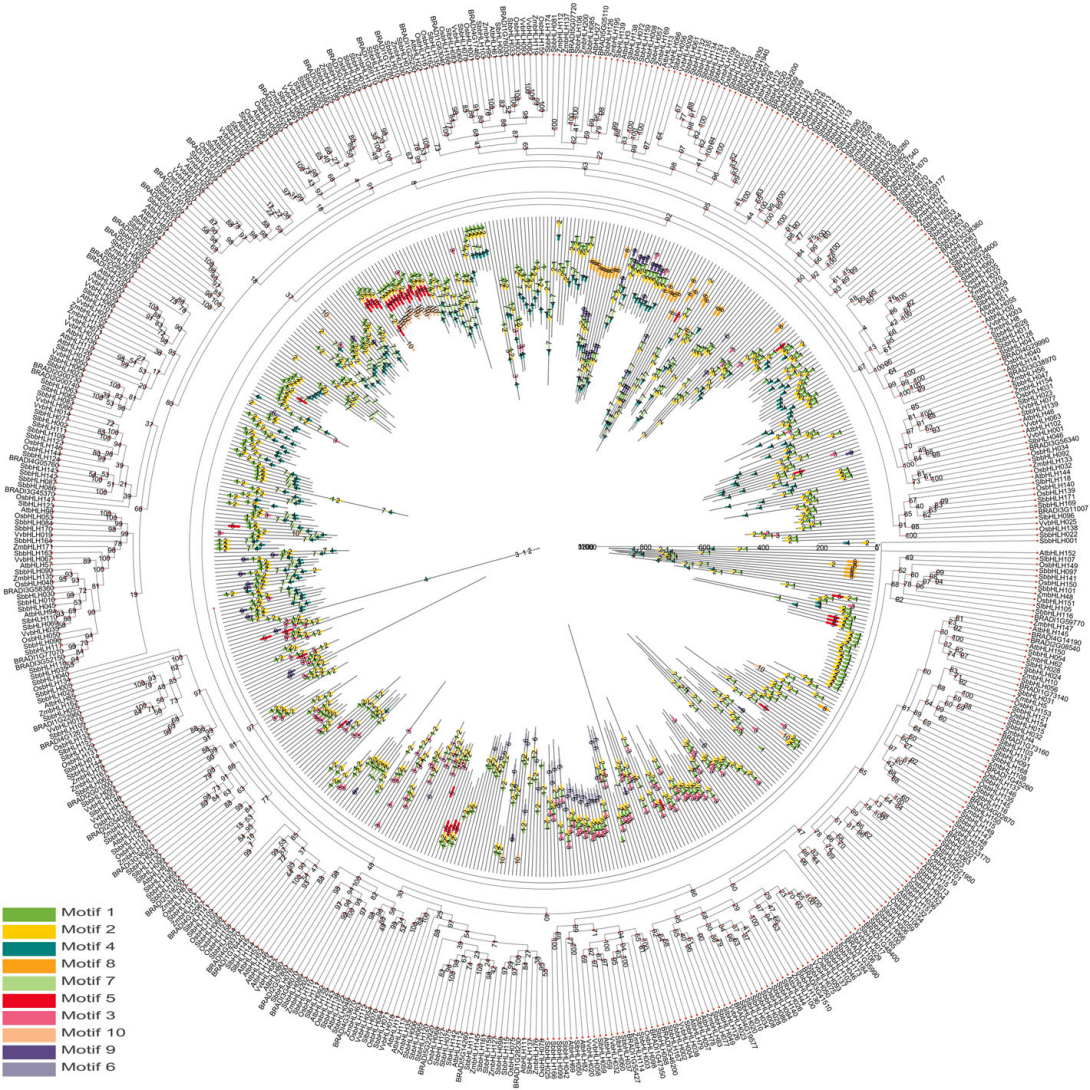
Phylogenetic relationship and motif composition of the bHLH proteins from *S. bicolor* with six different plant species (*Arabidopsis thaliana*, *Vitis vinifera*, *Solanum lycopersicum*, *Brachypodium distachyon*, *Oryza sativa subsp. indica*, *Zea mays*). Outer panel: An unrooted phylogenetic tree constructed using Geneious R11 with the NJ method. Inner panel: Distribution of the conserved motifs in bHLH proteins. The differently colored boxes represent different motifs and their positions in each bHLH protein sequence. The sequence information for each motif is provided in Additional File 2: Table S2.

To analyze the evolutionary relationship of the trihelix family of *bHLH* proteins among *S. bicolor* and six other plants (*A. thaliana*, *V. vinifera*, *S. lycopersicum*, *B. distachyon*, *O. sativa*, *Z. mays*), an unrooted NJ tree with 10 conserved motifs according to the MEME web server was constructed using the NJ method of Geneious R11 according to the protein sequences of 174 *SbbHLH* genes and the six other plants’ trihelix genes (Fig. 7, Additional file 2: Table S2). The detailed genetic correspondence is presented in Additional files 1 and 2: Tables S1 and S2. The distribution of *SbbHLH*s in the phylogenetic tree was relatively dispersed. As shown in Fig. 7, the SbbHLH proteins tended to gather with the bHLH proteins of *O. sativa* and *Z. mays*. With the exception of a few bHLH proteins, for example ZmbHLH8, ZmbHLH53, SbbHLH001, all other proteins of the six studied plants contained motifs 1 and 2. In addition, several motifs existed only in bHLH proteins of a few specific SbbHLH branches, such as motifs 5, 8 and 10. We also found that the bHLH proteins of *O. sativa*, *Z. mays* and *S. bicolor* on the same branch generally have similar motif compositions, and similar serial motifs tend to cluster in specific bHLH protein families. For example, serial motifs 1, 2, 5 and 10 tended to gather within group 6; and serial motifs 8, 9, 1, 2, 7 and 4 tended to gather within group 8. Thus, SbbHLH proteins may be more closely related to those of *O. sativa* and *Z. mays*.

### Expression patterns of *SbbHLH*s in several plant organs

**Fig. 8:**
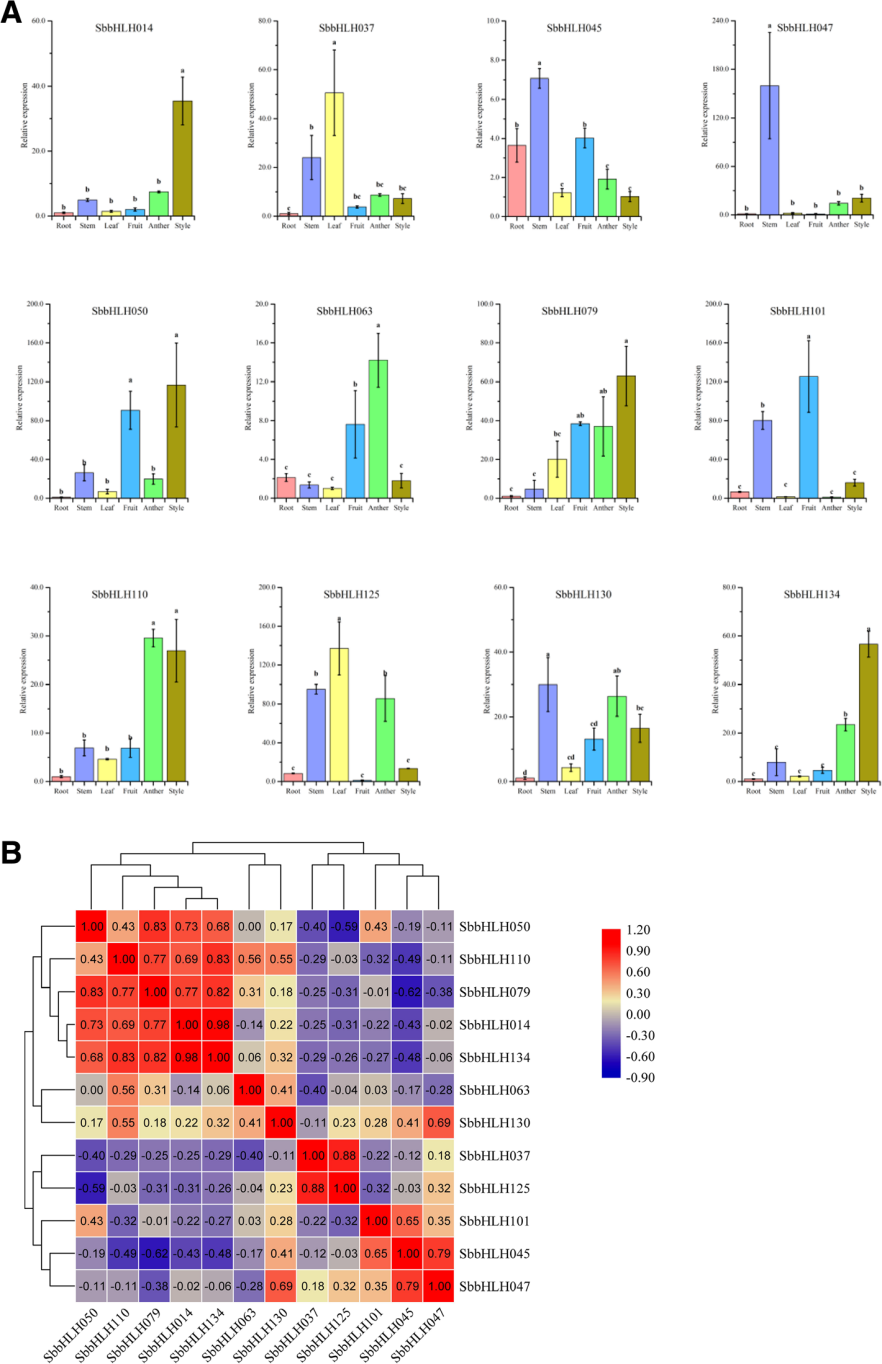
Tissue-specific gene expression of 12 *S. bicolor bHLH* genes and the correlation between their expression patterns. **a** Expression patterns of 12 *S. bicolor bHLH* genes in the anther, style, leaf, root, stem and fruit organs were examined by qRT-PCR. Error bars were obtained from three measurements. Lowercase letter above the bar indicates significant difference (α = 0.05, LSD) among the treatments. **b** Positive number: positively correlated; negative number: negatively correlated. Red numbers indicate a significant correlation at the 0.05 level

To investigate the potential roles of the *SbbHLH* genes, real-time PCR was used to detect the expression of 12 individual members of the gene family which were homologous to, or had close evolutionary relationships with *AtbHLH* genes with established functions. The accumulation of the transcriptional products of 12 *SbbHLH* genesfrom different subfamilies in six organs (anthers, styles, roots, leaves, fruit and stems) was evaluated (Fig. 8a). The results showed that some genes exhibited preferential expression in some tissues of *S. bicolor*. Most of the genes were expressed in all organs, and 4 genes (*SbbHLH014*, *SbbHLH050*, *SbbHLH079*, *SbbHLH134*) showed their highest expression level in the styles. Two genes (*SbbHLH063* and *SbbHLH110*) showed their highest expression level in the anthers, and the highest expression level of *SbbHLH037* and *SbbHLH125* was in the leaves. Three genes (*SbbHLH045*, *SbbHLH047* and *SbbHLH130*) showed highest expression in the *S. bicolor* stems, and the highest expression of *SbbHLH101* was found in fruit. In addition, correlations of *SbbHLH* expression among the six organs were studied (Fig. 8b). We found that the expression of different genes in the plant organs was significantly correlated, indicating that their roles may be synergistic. Most *SbbHLH* genes showed significant positive correlations; for example, we observed four genes—*SbbHLH050*, *SbbHLH079*, *SbbHLH014* and *SbbHLH134*—that had their highest expression in the styles, and were significantly positively correlated; they also showed significant positive correlations with *SbbHLH110*, which is most highly expressed in the anthers. However, four pairs of *SbbHLH* genes (*SbbHLH050* and *SbbHLH125*; *SbbHLH110* and *SbbHLH045*; *SbbHLH079* and *SbbHLH045*; *SbbHLH045* and *SbbHLH134*) were significantly negatively correlated.

### Expression patterns of *SbbHLH* genes in response to different treatments

**Fig. 9:**
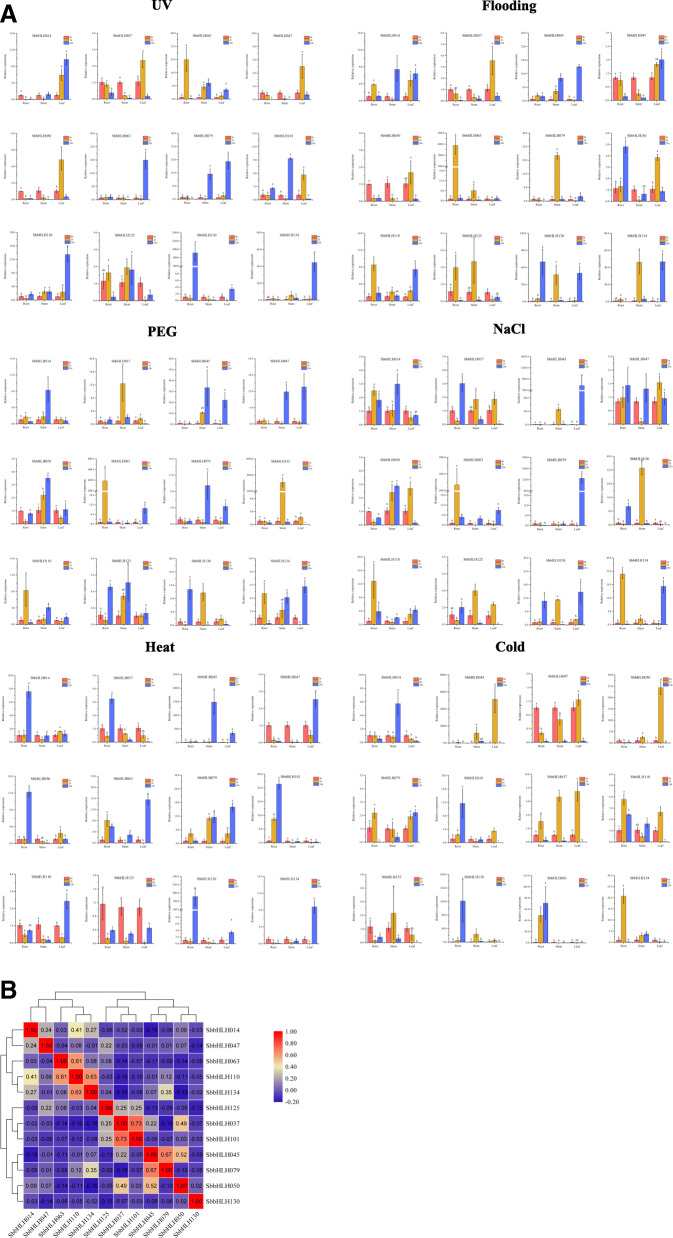
Gene expression of 12 *S. bicolor bHLH* genes in plants subjected to abiotic stresses (strong UV radiation, flooding, PEG, NaCl, heat and cold treatments) at the seedling stage. **a** Expression patterns of 12 *S. bicolor bHLH* genes in leaf, root and stem organs were examined by qRT-PCR. Error bars were obtained from three measurements. Lowercase letter above the bar indicates significant difference (α = 0.05, LSD) among the treatments. **b** Positive number: positively correlated; negative number: negatively correlated. Red numbers indicate a significant correlation at the 0.05 level

To further determine whether the expression of *SbbHLH* genes was influenced by different abiotic stresses, the expression of 12 *SbbHLH* members was examined under six abiotic stresses: strong ultraviolet (UV) radiation, flooding, polyethylene glycol (PEG), NaCl, heat and cold treatments. qRT-PCR analysis was performed to analyze the 12 *SbbHLH* genes’ expression patterns in roots, leaves and stems in response to the different treatments (Fig. 9a). Some of the *SbbHLH* genes were significantly induced or repressed by the different treatments. Expression of most of these genes was significantly altered in the early stage of the stress treatment (Fig. 9). Some *SbbHLH*s showed changes in expression with time or in different organs, depending on the stress. For example, under cold stress, *SbbHLH037* and *SbbHLH045* were first significantly upregulated, and then downregulated. *SbbHLH063* expression was significantly upregulated in the root, while it was significantly downregulated in the stem and leaf. Under flooding stress, *SbbHLH045* was significantly upregulated in the root, stem and leaf, but *SbbHLH050* was significantly downregulated. Interestingly, several genes showed opposing expression patterns under different treatments. The transcript levels of many *SbbHLH* genes, such as *SbbHLH063*, were upregulated in stems and leaves by the heat-stress treatment, but downregulated by the cold-stress treatment. Some other genes showed changes in specific organs. For instance, *SbbHLH014* responded significantly to heat treatment in the root. Furthermore, correlations between *SbbHLH* gene-expression patterns were observed (Fig. 9b). There were negative correlations among most *SbbHLH* genes. However, a few *SbbHLH* genes were significantly positively correlated, such as *SbbHLH110* and *SbbHLH063*/*SbbHLH134*, with *P* < 0.05 (Fig. 9b).

## Discussion

,Exploration of the *bHLH* gene family at the whole-genome level in any species, and the functional identification of some this family’s members, can provide theoretical support for the role of the *bHLH* gene family in the stress signal-transduction process. In this study, 174 *SbbHLH* genes were identified, and all of the encoded proteins showed obvious differences in structure, indicating high complexity. According to Atchley et al. [10] and Toledo-Ortiz et al. [15], we analyzed the DNA-binding ability of the basic region of *SbbHLH*s. The *SbbHLH* gene sequence can be divided into E-box binding genes and non-E-box binding genes (Additional file 1: Table S1). The E-box binding proteins can be subdivided into G-box binding proteins and non-G-box binding proteins [12, 15]. The basic domain of bHLH contains two essential amino acid residues, Glu-13 and Arg-16. If it contains only one of them, it will be classified as a non-E-box binding protein. The G-box binding protein contains three essential amino acid residues (His/Lys-9, Glu-13 and Arg-17) in the basic domain. If only Glu-13 and Arg-17 are present, it is classified as a non-G-box binding protein. In addition, if the number of basic amino acids is less than 4 in the basic domain, and it contains no or only one of Glu-13 and Arg-16, it will be classified as a non-DNA binding protein. These proteins are thought to have no ability to bind directly to DNA. In this study, 119 (68.4%) SbbHLHs were classified as E-box binding proteins: 99 (56.9%) as G-box binding proteins and 20 (11.5%) as non-G-box binding proteins; 30 (17.2%) members were classified as non-E-box binding genes. The remaining 25 (14.4%) members were not considered to have DNA-binding ability due to the lack of Glu-13 or Arg-16 in the alkaline region (Fig. 2, Additional file 1: Table S1). Similar to the reports of *O. sativa* (95, 56.9%) and *A. thaliana* (89, 60.5%), the highest proportion of *SbbHLH*s were G-box binding proteins [18]. Previous studies have found that some key amino acid residues play important roles in the binding of TFs to DNA and the formation of homodimers or heterodimers between bHLHs or bHLHs and other TFs [15, 70]. For example, His-6, Glu-10, and Arg-14 are related to DNA-binding activity, whereas Leu-25 and Leu-57 in the helical region determine whether bHLH TFs can form homodimers or heterodimers. In SbbHLHs, the conservation rates of Leu-25 and Leu-57 are 94.3 and 96.0%, respectively, which are lower than in *S. lycopersicum* (99, 97%) [47] and *Citrus reticulata* (100, 100%) [70]. Previous studies have found that the formation of such heterodimers can change or expand the diversity of molecular interactions, and generate new functions by identifying new DNA-binding sites [15,71]. As already noted, a bHLH protein can form a homodimer with itself or a heterodimer with other TFs, such as R2R3-MYBs, BAR1-BES1 and AP2 [72, 73,74].

,Based on the constructed phylogenetic tree, we identified at least one bHLH protein from *S. bicolor* in each subgroup of AtbHLHs, indicating that the time of differentiation of the bHLH family may have been earlier than that of *S. bicolor* and *A. thaliana*. The bHLH proteins within the reported subfamilies may play a fundamental role in the development, adaptation and evolution in dissimilar plant species, including peanut [51], tomato [48], Chinese cabbage [47], wheat [54], and *Carthamus tinctorius* [56]. Compared to *A. thaliana*, *SbbHLH* genes can be divided into 24 subfamilies and 1 orphan subfamily (UC), 4 more than *A. thaliana* and 3 more than *O. sativa*. Among them, group 15 (17, 9.8%) and group 18 (15, 8.6%) have more members, which is similar to the results for *A. thaliana* [3], and indicates that those *bHLH* gene groups may have undergone stronger partial differentiation in the long-term evolutionary process. However, there is no research to prove that this kind of differentiation is advantageous in the differentiation process of herbs and woody plants. Seven of the *SbbHLH*s did not have obvious clusters, so they were all classified into the UC group, and those genes all showed non-DNA binding activity, but still a great deal of variability in the base sequence. The gene-structure analysis revealed that *SbbHLH* genes in the same subfamily have similar gene structures, which not only supports our classification of the subgroups to a certain extent, but also indicates that all members of a subfamily are close in evolutionary terms (Fig. 3). At the same time, this does not rule out the loss of some independent introns during the long-term evolutionary development of plants, resulting in the loss of some introns in the domains of some bHLH members. For example, *SbbHLH153* has fewer introns than other members of the same family. Genes with few or no introns are considered to have lower expression levels in plants [4]. However, the compact gene structure may contribute to the rapid expression of genes in response to endogenous and/or exogenous stimuli [4]. Genome-replication events are considered to have occurred in the process of plant evolution, and the expansion of gene families and genome evolution mechanisms mainly depend on gene-replication events [5, 18,26]. The main replication modes are tandem repeats and fragment replication. These were identified in the *SbbHLH* genes. We discovered 13 tandem duplication events containing 20 *SbbHLH* genes (Fig. 48, Additional file 75: Table S3), especially on chromosomes 7 and 8. In addition, there were 42 pairs of segmental duplications of *SbbHLH* genes (Fig. 76, Additional file 77: Table S4). Therefore, segment duplication may make a higher contribution to the expansion of the bHLH family in *S. bicolor*. Nevertheless, since there were many duplication events in *S. bicolor*, it is lower than that of the dicotyledonous plants tomato and potato [78, 79]. Similar situations have been reported in studies of other monocotyledonous species [80]. However, the current conclusions cannot explain the significant differences between monocotyledons and dicotyledons.

Analysis of a gene’s expression profile can provide important clues to understanding its potential biological function. There are many members of the bHLH TF family with diverse functions, but the current research in plants is not particularly thorough, as it focuses mainly on the two model plants *A. thaliana* and *O. sativa*. The functions of bHLH TFs in other species still need to be explored. In this study, we used 12 *SbbHLH* genes with significant differences in clustering on the phylogenetic tree to study their responses to six abiotic stresses in different developmental organs, and found that almost all of the *bHLH* TF genes have significant differential expression (more than 2-fold difference). For example, under salt stress, 10 *SbbHLH*s were upregulated in leaves, 7 were upregulated in roots, and 8 were upregulated in stems. The expression pattern results indicate that bHLH TFs participate in a complex cross-regulatory network. *SbbHLH079* and *SbbHLH045* were responsive, at the same time, to PEG, NaCl and UV treatments, indicating synergistic or antagonistic regulation under a variety of adverse conditions. Further research is needed to explore the relationship between these genes. Interestingly, most of the *SbbHLH* genes showed significant negative regulation in the expression heat map. If we consider expression patterns and complex protein interactions, then we can suggest that a network of feedback mechanisms coordinates the expression of multiple genes. In addition, flowers and fruit, as plant reproductive organs, are the main structures in all angiosperms [81]. In this study, we explored the expression of 12 *bHLH* genes in the anthers and styles of *S. bicolor* flowers, as well as in the main organs of plants at the filling stage. Studies have shown that bHLH TFs play an important role in the development of flowers and fruit. The expression levels of *SbbHLH134* and *SbbHLH110* in the anther and style were significantly higher than in roots, stems, leaves and fruit, whereas *SbbHLH101* showed significantly higher expression in fruit at the filling stage (Fig. 8a). Therefore, we speculate that *SbbHLH134*, *SbbHLH110* and *SbbHLH101* may also regulate flower and fruit development in *S. bicolor*. However, the specific functions still need to be analyzed through in-depth experiments. In summary, these results reveal the functions and regulation of some bHLH TFs.

## Conclusion

In summary, we provided the systematic genome-wide analysis of the *bHLH* gene family in *S. bicolor.* A total of 174 *SbbHLH* genes/proteins were characterized and divided into 24 groups. Furthermore, protein motifs and gene structures of the *SbbHLH*s within the subfamilies were prone to be the similar, which supported the classification predicted. The distribution of the 174 *SbbHLH* genes on 10 *S. bicolor* chromosomes was uneven. We found that gene-replication events may have produced some *SbbHLH* genes, with tandem duplication contributing more to the expansion of the *SbbHLH* gene family than segmental duplication. The qRT-PCR results showed that the 12 studied *SbbHLH*s were all affected by abiotic stresses, and their expression during the development of flowers and fruit was studied. It is speculated that *SbbHLH134*, *SbbHLH110* and *SbbHLH101* also regulate flower and fruit development in *S. bicolor*. Taken together, the results and information described in this work provide a good basis for further investigation of the biological functions and evolution of *bHLH* genes in *S. bicolor*.

## Methods

### Gene identification

,We downloaded the complete *S. bicolor* genome sequence from the Ensembl Genomes website (http://ensemblgenomes.org/). Based on two BLASTp searches [82,83], bHLH family members were identified. First, with BLASTp (score value ≥100 and e-value ≤1e- 10), all possible bHLH proteins were identified from the *S. bicolor* genome referring to trihelix protein sequences of *A. thaliana*. Second, the Hidden Markov Model (HMM) profile consistent with the trihelix domain was obtained from the Pfam protein family database (http://pfam.sanger.ac.uk/). We used both HMMER3.0 (default parameters) with a cutoff of 0.01 (http://plants.ensembl. org/hmmer/index.html) [84] and SMART (http://smart.embl-heidelberg.de/) [85,86] to ascertain the presence of the bHLH domain, and to further verify the results. In addition, the basic features of the trihelix proteins of the *SbbHLH* gene family were identified: coding sequence length, pI, protein molecular mass, and subcellular localization, from the ExPasy website (http://web.expasy.org/protparam/).

### *bHLH* gene structure

,The bHLH domain sequences of the characterized SbbHLH proteins were used to create multiple protein sequence alignments using ClustalW with default parameters [87]. The deduced amino acid sequences in the bHLH domains were then adjusted manually using GeneDoc software. We used Gene Structure Display Server (GSDS: http://GSDS.cbi.pku.edu.cn) [88] to analyze the constituents of the exons/introns of the *SbbHLH* genes. We used MEME to analyze the motifs of SbbHLH proteins, (http://meme-suite.org/tools/meme) [89,90]. The optimized parameters were as follows: number of repetitions, any; the maximum number of motifs, 10; and the optimum width of each motif, between 6 and 200 residues [83,90,91].

### Chromosomal distribution and gene duplication

All *SbbHLH* genes were mapped to *S. bicolor* chromosomes based on physical location information from the database of the *S. bicolor* genome using Circos [92]. The Multiple Collinearity Scan toolkit (MCScanX) was adopted to analyze the gene-duplication events, with the default parameters [93]. We analyzed homology of the *bHLH* genes between *S. bicolor* and the other six plants (*A. thaliana*, *V. vinifera*, *S. lycopersicum*, *B. distachyon*, *O. sativa* subsp. *indica*, *Z. mays*) using Dual Synteny Plotter (https://github.com/CJ-Chen/TBtools). Non-synonymous (ka) and synonymous (ks) substitutions of each duplicated *bHLH* gene were calculated using Ka/Ks-Calculator 2.0 [94].

### Phylogenetic analysis and classification of *SbbHLH* gene family

According to the classification of *AtbHLH*s, all of the identified *SbbHLH* genes were divided into groups. The phylogenetic trees were inferred using the NJ method of MEGA X via Geneious R11 with BLOSUM62 cost matrix, the Jukes–Cantor model, global alignment with free end gaps and bootstrap value of 1000. The full-length amino acid sequences of the bHLH proteins (Additional file 1: Table S1) derived from *A. thaliana*, *V. vinifera*, *S. lycopersicum*, *B. distachyon*, *O. sativa* subsp. *indica*, and *Z. mays* (UniProthttps://www.uniprot.org/), combined with newly identified *SbbHLH*s, were used for phylogenetic analysis.

### Plant materials, growth conditions, and abiotic stress in *S. bicolor*

*Sorghum bicolor* cv. Hongyingzi*,* a typical cultivated variety, was used throughout the study. Since 2019, ‘Hongyingzi’ has been grown in the greenhouse of Guizhou University. *S. bicolor* was grown in pots filled with soil and vermiculite (1:1) in a growth room with a 16 h/25 °C day and 8 h/20 °C night regime, and a relative humidity of 75%. We collected the stems, roots, leaves, fruit, anthers and styles separately from five plants with good growth and similar growth conditions, and quickly placed them in liquid nitrogen for storage at -80 °C pending further use. To investigate gene-expression patterns in response to various stresses, several *SbbHLH* genes were selected for further analysis. *S. bicolor* plants were subjected to the following abiotic stress treatments at the seedling stage (21 days): salt (5% NaCl), water flooding (whole plant), drought (30% PEG6000), UV exposure (70 μW/cm^2^, 220 V, 30 W), high temperature (40 °C), and low temperature (4 °C); each stress treatment was performed with five replicates, and qRT-PCR analysis was carried out after sampling at 2 h and 24 h, respectively. The collected samples were stored at -80 °C for further analysis.

### Total RNA extraction, cDNA reverse transcription and qRT-PCR analysis

,Total RNA of each sample was extracted with a plant RNA extraction kit (TaKaRa) and used for cDNA library construction. The sequencing was performed in an Illumina GAII sequencer following the manufacturer’s instructions [90,6]. Gene-expression analysis was performed by qRT-PCR, with primers designed by Primer 5.0 (Additional file 91: Table S6). We used the *GAPDH* (glyceraldehyde-3-phosphate dehydrogenase) gene, which was stably expressed at each growth stage in almost all tissues, as an internal control [95]. The qRT-PCR with SYBR Premix Ex Taq II (TaKaRa) was repeated at least three times and the data were analyzed using the 2^− (ΔΔCt)^ method [96].

### Statistical analysis

Analysis of variance (ANOVA) was performed with JMP6.0 software (SAS Institute), and compared with least significant difference (LSD) at the 0.05 and 0.01 levels. The histogram was drawn with Origin 8.0 software (OriginLab).

## Supplementary Information

**Additional file 1 Table S1.** List of the 174 *S. bicolor bHLH* genes identified in this study.

**Additional file 2 Table S2.** Analysis and distribution of the conserved motifs in *S. bicolor* bHLH proteins.

**Additional file 3 Table S3.** Tandem duplication events of *S. bicolor bHLH* genes.

**Additional file 4 Table S4.** The 42 pairs of segmental duplications in *S. bicolor bHLH* genes.

**Additional file 5 Table S5.** One-to-one orthologous genes relationships between *S. bicolor* and other plants.

**Additional file 6 Table S6.** Primer sequences for qRT-PCR.

## Data Availability

The entire *Sorghum bicolor* genome sequence information was from the Ensembl Genomes website (http://ensemblgenomes.org/). The *Sorghum bicolor* materials (Hongyingzi) used in the experiment were supplied by Prof. Cheng Jianping of Guizhou University. The datasets supporting the conclusions of this article are included in the article and its Additional files.

## Abbreviations

bHLH: helix-loop-helix
SbbHLH: *Sorghum bicolor* bHLH
GAPDH: Glyceraldehyde-3-phosphate dehydrogenase
qRT-PCR: Quantitative real-time polymerase chain reaction
TF: Transcription factor
AtbHLH: *Arabidopsis thaliana* bHLH
HMM: Hidden Markov Model
pI: Isoelectric point
LG: Linkage group
